# Comparison of accidental findings of brain magnetic resonance imaging of patients with obsessive-compulsive disorder and healthy controls

**DOI:** 10.1186/s12888-023-05393-5

**Published:** 2023-12-01

**Authors:** Olga Bayar Kapıcı, Yaşar Kapıcı, Atilla Tekin

**Affiliations:** 1 Department of Radiology, Adıyaman Training and Research Hospital, Adıyaman, Turkey; 2 Department of Psychiatry, Adıyaman University Medical Faculty, Adıyaman, Turkey

**Keywords:** Obsessive-compulsive disorder, Brain magnetic resonance imaging, Neurodevelopmental

## Abstract

**Background:**

Abnormalities in brain magnetic resonance imaging (MRI) have been reported in drug-naive and chronic patients with obsessive-compulsive disorder (OCD). The Fazekas scale is a method used to categorize and grade the severity of white matter hyperintensities (WMH) in brain MRI. These lesions can be indicative of various neurological conditions, particularly small vessel disease or cerebrovascular pathology.

**Methods:**

Brain MRIs of patients followed up with the diagnosis of OCD were retrospectively analyzed. 58 OCD (36 females, 22 males) and 58 healthy controls (HC) (30 females, 28 males) were included in the study. Age, gender, and brain MRI findings of the participants were recorded.

**Results:**

The mean ages of the OCD and HC groups were 33.4 ± 10.6 and 35.9 ± 9.3. There was no difference between the groups in terms of mean ages and gender distribution (p = 0.180 and p = 0.260, accordingly). Generalized cerebral atrophy was more common in patients with OCD than in HC (p = 0.008). Fazekas grade 1 was detected in 17.2% of the patients with OCD and 1.7% of HC. Accordingly, it was significantly more common in Fazekas grade 1 OCD patients (p = 0.002). Fazekas grade 2 was detected in only 2 patient with OCD. CVI was present in 20.7% of the patients with OCD and 1.7% of HC. There was a significant difference between the groups regarding CVI (p = 0.001). Ethmoidal thickening was more common in patients with OCD than in HC (p = 0.004). The YBOCS scores and ages of OCD patients with Fazekas grade 1 and 2 were significantly higher than those of patients with Fazekas grade 0. Likewise, the YBOCS scores and ages of OCD patients with generalized cerebral atrophy were significantly higher than those of patients without atrophy.

**Conclusion:**

It is understood from the present study’s findings that CVI, a neurodevelopmental malformation, is more common in patients with OCD. Due to the potential relationship of this anomaly with neuronal migration, it would be appropriate to pay attention to OCD symptoms in individuals with CVI and to perform white matter examination on brain imaging. In future studies, Fazekas grade can be evaluated in drug-naive OCD patients, and data on the pre-disease period can be obtained.

## Introduction

Obsessive-compulsive disorder (OCD) is a psychiatric disorder that leads to severe and chronic disability involving disturbing obsessions and compulsions that reduce mental tension caused by obsessions. Obsessions are repetitive, unwanted, illogical, and stressful thinking patterns. Compulsions are automatic actions or mental habits that neutralize the anxiety associated with obsessions [1]. The life-long occurrence frequency of OCD is predicted to vary between 2 and 3%. In most patients, this disorder begins in childhood or puberty. Nearly half of these cases, signs continue throughout the adult years. Patients struggling with OCD may suffer severe difficulties and impairments in vocation, domestic, and interpersonal life, often leading to a diminished life standard [2]. In addition to the negative effects of OCD on the lives of particular sufferers, the cost of OCD at the community tier is also very high. In 1996, the World Health Organization listed OCD as one of the ten major reasons for disability in the world [3].

The etiopathogenesis of OCD is not yet fully understood. Brain scanning investigations and neuropsychological evidence show that several brain structures are involved in the development of OCD. It is thought that the orbitofrontal cortex, anterior cingulate cortex, basal ganglia, and thalamus are usually impacted in OCD [4]. Various findings were observed in researches on the caudate nucleus and putamen volumes. While one study found an increased caudate nucleus volume, another found no difference in the caudate nucleus and putamen volumes between patients with and without OCD [5, 6]. Birth trauma, head trauma, epileptic disorders, Parkinson’s disease, Huntington’s disease, Sydenham’s chorea, progressive supranuclear palsy, Gilles de la Tourette’s syndrome, frontal lobe tumors, neuroacanthocytosis, neonatal hypoxia, bilateral caudate infarcts, and carbon monoxide and manganese poisoning may induce OCD symptoms [7]. Therefore, brain magnetic resonance imaging (MRI) can be requested in patients with OCD symptoms.

The Fazekas scale is a method used to categorize and grade the severity of white matter hyperintensities (WMH) in brain imaging studies, such as MRI. It’s named after László Fazekas, a Hungarian neurologist who developed the scale. These lesions can be indicative of various neurological conditions, particularly small vessel disease or cerebrovascular pathology. The Fazekas scale typically involves two grades: Fazekas Score 0: This indicates the absence of significant white matter hyperintensities. Fazekas Score 1, 2, or 3: These scores reflect increasing severity of WMH. Higher Fazekas scores are often associated with a higher risk of cognitive impairment [8].

Although imaging studies have shown that there are anatomical differences in many brain regions in OCD patients, it has also been suggested that incidental anatomical differences can be discovered in these patients. Therefore, it has been aimed the detect incidental changes in OCD. We thought that these incidental changes might help future studies to discover the unknowns of OCD. In this study, abnormalities such as brain atrophy, gliotic foci, cavum septum pellucidum, cavum vergae, and cavum velum interpositium (CVI), cysts, and WMH of the patients with OCD and healthy controls (HC) were examined and compared. The obtained data provide valuable insight into the literature on brain abnormalities in patients with OCD. This study may be useful in etiological evaluation in terms of examining the relationship between OCD and brain pathologies. In future studies with drug-naive OCD patients, brain MRI images can be compared before and after the disorder.

## Materials and methods

### Study design

Brain MRIs of patients followed up with the diagnosis of OCD in the Psychiatry Clinic of Adıyaman Training and Research Hospital between 01/01/2018 and 01/09/2022 were retrospectively analyzed. Adıyaman University Ethics Committee approved this research (Date of Decision: 15/11/2022, IRB Number: 2022/8–27).

### Study group and data collection

**Fig. 1:**
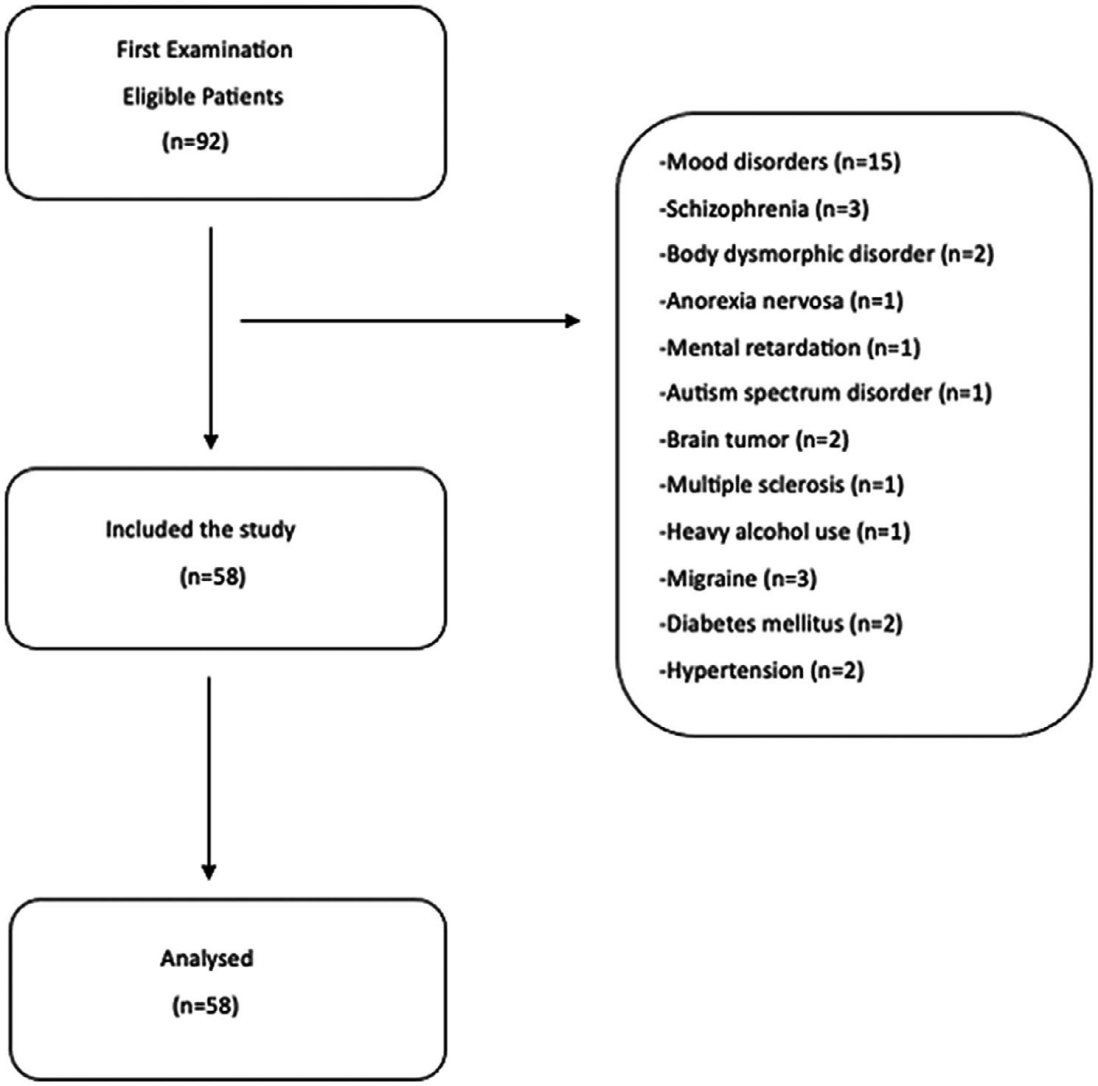
Flow-chart illustration of the study’s sample

Ninety two patients with OCD were identified and 23 of them were eliminated primarily because of serious comorbid psychiatric disorders. Of the 23 excluded patients, 15 had comorbid mood disorders, 3 had schizophrenia, 2 had body dysmorphic disorder, 1 had anorexia nervosa, 1 had mental retardation, and 1 had autism spectrum disorder. Within the scope of the study, 69 patients who were followed up with the diagnosis of OCD without any other severe psychiatric comorbidity (such as schizophrenia, bipolar disorder, alcohol or substance use disorder, mental retardation) were examined. Brain MRI may be requested for differential diagnosis or to rule out additional neurological diseases in patients with OCD. Some OCD patients may apply to neurology and psychiatry with complaints such as headache and dizziness. In such cases, a brain MRI examination is requested. Additionally, brain MRI may be performed in OCD patients in case of accompanying psychotic symptoms. Of 69 patients with OCD, twelve patients were excluded from the study due to comorbidities [brain tumor (n = 2), multiple sclerosis (n = 1), heavy alcohol use (n = 1), migraine (n = 3), diabetes mellitus (n = 2), hypertension (n = 2)]. Flow-chart illustration of the study’s sample was shown in Fig. 1. As the HC group, people who were requested to have a brain MRI examination for reasons such as non-migraineous headache and vertigo and whose brain MRI was reported as normal were selected. It was noted that the HC group did not have a previous history of psychiatric disease.

### Sociodemographic features

Age and gender characteristics of all participants were obtained by scanning the medical database of Adıyaman Training and Research Hospital.

### Yale Brown Obsessive-Compulsive Scale

Regardless of the kind of obsessions and compulsions, Goodman et al. created it in 1989 to assess the severity of OCD [9]. Ten items are rated between 0 and 4 on the clinician’s scale. Karamustafalolu et al. conducted a validity and reliability evaluation of this measure in Turkey. Yale Brown Obsessive-Compulsive Scale-Total Score **(**YBOCS) cut-off points 0–7 non-clinical level; 8–15 light; 16–23 moderate intensity; 24–31 severe; 32–40 extremely severe [10].

### Brain MRI examination

**Fig. 2:**
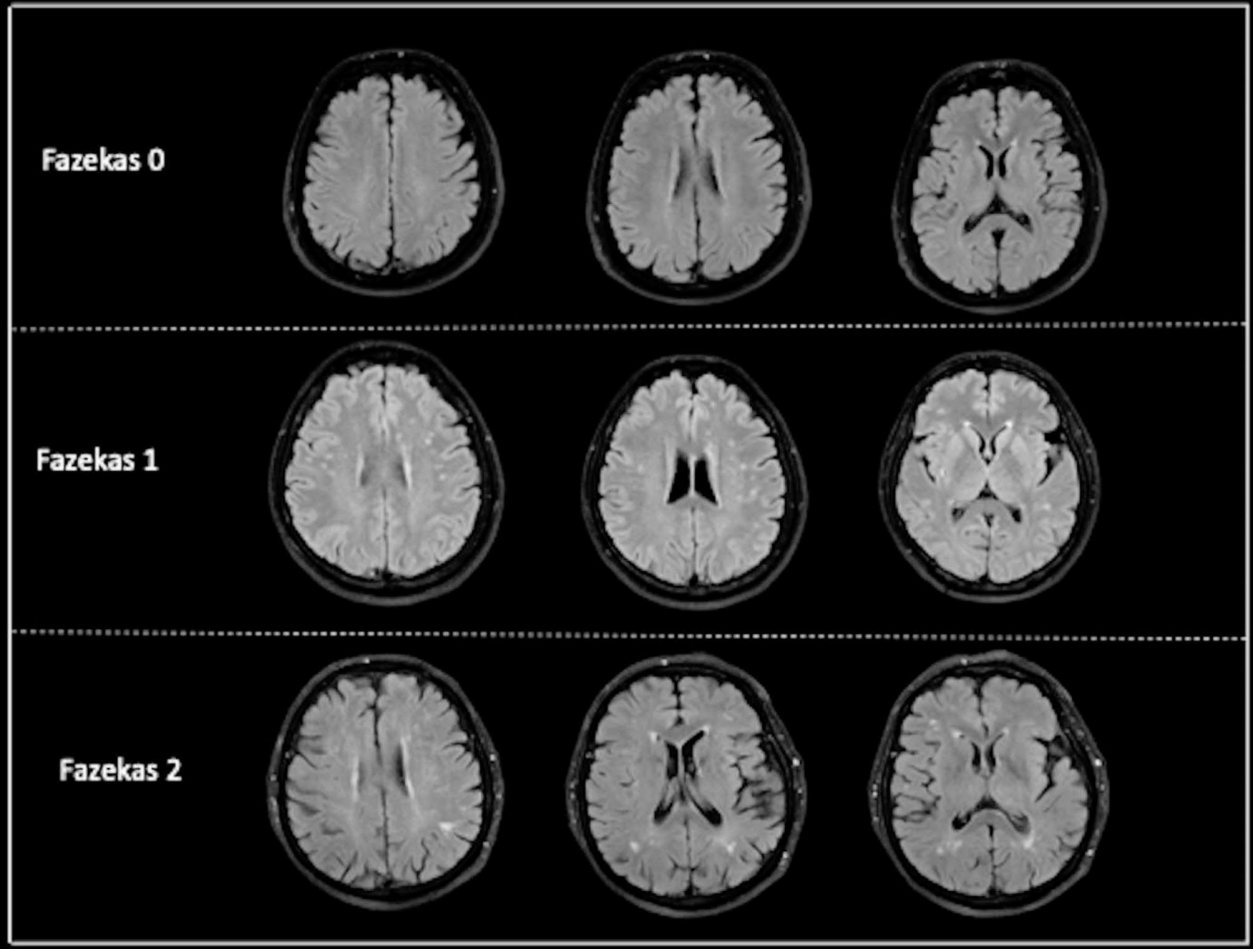
Fazekas grading on brain MRI

The MRI examination was undertaken on the Philips Achieva MR device (Philips Medical Systems, Best, Netherlands) with a 1.5 Tesla magnetic field strength applied with a head coil. From the T1 FLAIR-weighted images taken in the sagittal plane, the cross-section through the cranial midline where the mass intermedia can be seen was examined. [time to repeat (TR): 1665 ms, time to echo (TE): 20 ms, FOV: 220 × 230, slice thickness: 5 mm, matrix: 292 × 214, NSA: 1, gap :1 mm, voxel: 0.75 × 1.07 × 5, slices : 24 sections]. Images were evaluated at Philips Achieva Rev R5 v30-rev.02 workstation and our hospital’s PACS system. The assessment was conducted on brain MRI T1-weighted axial, T2-weighted axial, FLAIR axial, and T2-weighted coronal and T1-weighted sagittal images. Brain MRI findings, age, and gender information were noted. Fazekas scale was employed to determine the degree of brain aging. The Fazekas scale was made in accordance with WMH specifications. Fazekas grade 0 (none), 1 (spot foci), 2 (beginning grouping of foci), or 3 (widely clustered regions) [11]. Fazekas grading on brain MRI was shown in Fig. 2.

### Statistical analysis

IBM SPSS Statistics Mac version 26.0 (IBM SPSS Inc., Chicago, IL, USA) was used to complete statistical analysis. Continuous variables were given as mean ± standard deviation, and categorical variables were given as percentages (%). Kurtosis, skewness values, and the Shapiro-Wilk test were performed to determine whether continuous variables were normally distributed. Student-t test was used to compare the mean ages of the groups. The Chi-square and Fischer’s Exact tests were used to compare categorical variables. The Mann-Whitney U test was used for the comparison of non-normally distributed parameters. The level of significance was adjusted using Bonferroni’s corrections in multiple comparisons in order to minimize the risk of type I error [12].

## Results

Fifty eight OCD (36 females, 22 males) and 58 HC (30 females, 28 males) were included in the study. The comparison of the sociodemographic characteristics of the OCD and HC groups is shown in Table 1. The mean ages of the OCD and HC groups were 33.4 ± 10.6 and 35.9 ± 9.3. There was no difference between the groups in terms of mean ages and gender distribution (p = 0.180 and p = 0.260, accordingly).

**Table 1:** Comparison of sociodemographic features of patients with obsessive-compulsive disorder and healthy controls

	OCD (n = 58) M ± SD or n (%)	HC (n = 58) M ± SD or n (%)	*p*
Age (years)	33.4 ± 10.6	35.9 ± 9.3	0.180^a^
Gender			0.260^b^
Female	36 (62.1)	30 (51.7)	
Male	22 (37.9)	28 (48.3)	

OCD, Obsessive-compulsive disorder; HC, Healthy controls

^a^Independent t test was used

^b^Chi-square test was used. p < 0.05 was accepted as statistically significant

The frequency of accidental MRI findings of the groups was compared in Table 2. Generalized cerebral atrophy was more common in patients with OCD than in HC (p = 0.008). Fazekas grade 1 was detected in 17.2% of the patients with OCD and 1.7% of HC. Accordingly, it was significantly more common in Fazekas grade 1 OCD patients (p = 0.002). Fazekas grade 2 was detected in only 2 patient with OCD. CVI was present in 20.7% of the patients with OCD and 1.7% of HC. There was a significant difference between the groups regarding CVI (p = 0.001). Ethmoidal thickening was more common in patients with OCD than in HC (p = 0.004). Pituitary macroadenoma, neuroglial cyst, parotid liposubstitution, and demyelination plaque were detected only in patients with OCD. Tornwalt’s Cyst was detected in only 1 HC.

**Table 2:** Comparison of incidental MRI findings between patients with obsessive-compulsive disorder and healthy controls

		OCD (n = 58) N/%	HC (n = 58) N/%	χ^2^	*p*
Atrophy	Generalized Cerebral	9 / 15.5	1 / 1.7	7.004	**0.008**
Fazekas	Grade 1	10 / 17.2	1 / 1.7	10.398	0.002*
	Grade 2	2 / 3.4	0 / 0		
Cavum Septum Pellucidum		5 / 8.6	1 / 1.7	2.812	0.094
Cavum Vergae		3 / 5.2	1 / 1.7		0.618*
Cavum Veli Interpositi		12 / 20.7	1 / 1.7	10.482	**0.001**
Ethmoidal Thickening		29 / 50	14 / 24.1	8.315	**0.004**
Maxillary Thickening		7 / 12.1	13 / 22.4	2.175	0.140
Sphenoid Thickening		3 / 5.2	2 / 3.4		1.000*
Frontal Thickening		1 / 1.7	2 / 3.4		1.000*
Retention Cyst		11 / 19	13 / 22.4	0.210	0.647
Septal Deviation		20 / 42.5	18 / 36	2.336	0.264
Adenoid Hypertrophy		10 / 17.2	9 / 15.5	0.063	0.802
Tornwalt’s Cyst		0 / 0	1 / 1.7		1.000*
Nonspesific Gliotic Foci		4 / 6.9	3 / 6		1.000*
Demyelination Plaque		1 / 1.7	0 / 0		1.000*
Mastoiditis		2 / 3.4	2 / 3.4		1.000*
Partial Empty Sella		2 / 3.4	1 / 1.7		1.000*
Pituitary Macroadenoma		1 / 1.7	0 / 0		1.000*
Neuroglial Cyst		1 / 1.7	0 / 0		1.000*
Parotid Liposubstitution		1 / 1.7	0 / 0		1.000*
Arachnoid Cyst		4 / 6.9	1 / 1.7		0.364*

Chi-square test was used. p values < 0.05 being considered significant

*Fischer’s exact test

Comparison of incidental MRI findings in OCD patients according to gender is shown in Table 3. Accordingly, although CVI was more common in females, it was not found to be statistically significant according to Bonferroni correction.

**Table 3:** Comparison of incidental MRI findings between genders in patients with obsessive-compulsive disorder

		Male (n = 22) N/%	Female (n = 36) N/%	χ^2^	*p*
Atrophy	Generalized Cerebral	4 / 18.2	5 / 13.9	0.192	0.661
Fazekas	Grade 1	4 / 18.2	6 / 16.7	0.955	0.749 N/A
	Grade 2	0 / 0	2 / 5.6		
Cavum Septum Pellucidum		2 / 9.1	3 / 8.3	2.812	1.000*
Cavum Vergae		3 / 13.6	0 / 0		N/A
Cavum Veli Interpositi		1 / 4.5	11 / 30.6	5.630	0.021*
Ethmoidal Thickening		13 / 59.1	16 / 44.4	1.172	0.279
Maxillary Thickening		2 / 9.1	5 / 13.9		0.698*
Sphenoid Thickening		2 / 9.1	1 / 2.8		0.551*
Frontal Thickening		1 / 4.5	0 / 0		0.379*
Retention Cyst		7 / 31.8	4 / 11.1	3.810	0.051
Septal Deviation		20 / 42.5	18 / 36	2.336	0.264
Adenoid Hypertrophy		4 / 18.2	6 / 16.7	0.022	0.882
Nonspesific Gliotic Foci		2 / 9.1	2 / 5.6	0.266	0.630*
Demyelination Plaque		1 / 4.5	0 / 0		1.000*
Mastoiditis		1 / 2.8	1 / 4.5	0.128	1.000*
Partial Empty Sella		0 / 0	2 / 5.6		1.000*
Pituitary Macroadenoma		1 / 4.5	0 / 0		1.000*
Neuroglial Cyst		1 / 4.5	0 / 0		1.000*
Parotid Liposubstitution		0 / 0	1 / 2.8		1.000*
Arachnoid Cyst		2 / 9.1	2 / 5.6	0.266	0.630*

Chi-square test was used. p values < 0.05 being considered significant

*Fischer’s exact test

Comparison of YBOCS scores and ages of patients with OCD according to the incidental MRI findings were shown in Table 4. Accordingly, the YBOCS scores and ages of OCD patients with Fazekas grade 1 and 2 were significantly higher than those of patients with Fazekas grade 0. Likewise, the YBOCS scores and ages of OCD patients with generalized cerebral atrophy were significantly higher than those of patients without atrophy.

**Table 4:** Comparison of YBOCS scores and ages of patients with obsessive-compulsive disorder according to the incidental MRI findings

	Fazekas (Grade 0) (n = 46) med (min-max)	Fazekas (Grade 1–2) (n = 12) med (min-max)	*p*
YBOCS	28 (19–34)	32 (28–36)	**< 0.001**
Age	30 (18–59)	42.5 (31–54)	**< 0.001**
	**Non-Atrophy**	**Generalized Cerebral Atrophy**	
YBOCS	28 (19–36)	31 (24–34)	**0.034**
Age	30 (18–59)	39 (31–54)	**0.007**
	**Non-Cavum Veli Interpositi**	**Cavum Veli Interpositi**	
YBOCS	29 (19–36)	28.5 (20–33)	0.582
Age	31 (18–59)	34 (18–54)	0.558

The Mann-Whitney U test was used. p < 0.05 was accepted as statistically significant

YBOCS, The Yale-Brown Obsessive-Compulsive Scale

## Discussion

The findings obtained in this study can be listed as follows: (i) Fazekas grade 1 was detected more frequently in patients with OCD, (ii) CVI was detected more frequently in patients with OCD, (iii) Although not statistically significant, brain atrophy was numerically higher in patients with OCD.

Neuroimaging studies in OCD dating back to the 1980s showed significant differences between patients and healthy controls [13]. Several neurobiological models involving various substructures of the frontal-striatal-thalamic-cortical network have been proposed for the etiopathogenesis of OCD [14]. These models include the hypothesis that neurodevelopmentally mediated mesh dysplasia can lead to ventral prefrontal-striatal abnormalities [15]. Some authors have suggested that other structures, such as the brainstem, amygdala, or corpus callosum, are also involved [16]. In the last two decades, modern imaging techniques have contributed significantly to our understanding of neuropsychiatric disorders such as OCD.

Although the volumes of brain structures were not measured in the MRI studies performed by Garber et al., it was concluded that there was no structural abnormality in patients with OCD. Researchers compared OCD patients with a family history with those without and with normal controls. They reported more abnormalities in the anterior cingulate gyrus on T1-weighted MRI in the first group. They found a positive correlation between the severity of symptoms and orbitofrontal cortex right-left asymmetry in this group [17]. Many MRI studies have shown abnormalities in caudate nucleus volume in patients with OCD, but the studies have not been consistent in this aspect [4]. Similarly, Jenike et al. observed a loss of asymmetry, a decrease in total cerebral and cerebellar white matter, and an increase in total cerebral cortical volume in patients with OCD compared to controls [18]. Aylward et al. found no difference in caudate nucleus volume between the OCD group and controls [6].

**Fig. 3:**
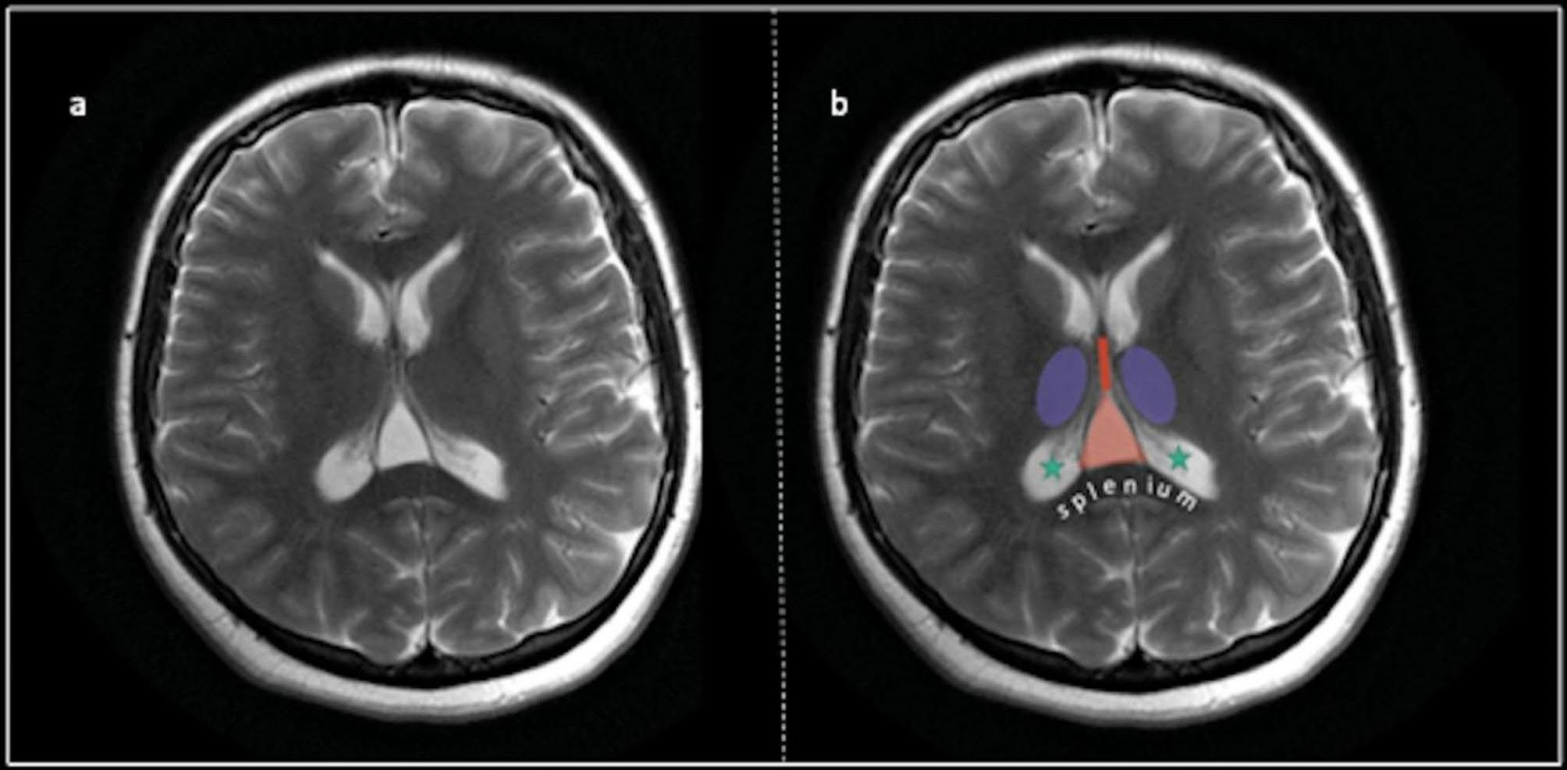
Cavum veli interpositi on brain MRI. *Notes*: Purple: Thalamus; Red: Third ventricle; Triangle-shaped orange area: CVI; Green stars: Lateral ventricles

One of the interesting finding of the present study is that CVI is more frequent in patients with OCD than healthy controls. To our knowledge, studies reporting the prevalence of CVI in OCD patients are very few. Some studies have reported that CVI is more common especially in neurodevelopmental and neurodegenerative diseases. The velum interpositium is located below the fornix and hippocampal commissure. In front of the velum interpositium are the interventricular foramina and the roof of the third ventricle, and behind there are splenium of corpus callosum and the habenular commissure. Inferior of the velum interpositium there are tela choroidea of the third ventricle and the internal cerebral veins. Thalami is located lateral to the CVI. Tela choroidea is an extension of the piamaeter and is a densely vascularized, thin soft tissue structure. The velum interpositium is the double-layered tela choroidea of the third ventricle, and the fluid-filled structure is called CVI. CVI and neighbouring structures were presented on brain MRI on Fig. 3. It is unclear whether CVI is a developmental disorder or a variant of normal development [19]. Major CVI has been reported in epilepsy, schizophrenia, mental retardation, and hydrocephalus [20]. CSP, CV, and CVI are considered permanent primitive structures as they are considered normal in fetal development. It has been hypothesized that these midline anatomical abnormalities may affect neuronal migration into the limbic system. Therefore, it can cause cognitive, emotional, and behavioral symptoms [21]. Detection of these anomalies more frequently in patients with schizophrenia has led to investigation of these malformations in other psychiatric diseases [22]. Although OCD is thought to be among the neurotic disorders, there are cases where insight is impaired, and obsessions reach the level of delusions in OCD [23]. Neurodevelopmental anomalies are thought to be more common in pediatric-onset, psychotic, and treatment-resistant cases of OCD [24].

The Fazekas scale is a grading system applied according to the frequency and distribution of WMH in brain MRI. The frequency of WMH increases with age and indicates the brain’s aging. It has been reported that cognitive functions deteriorate as the Fazekas grade increases. Fazekas grade has been found to predict cognitive performance in Alzheimer’s patients [25]. WMH has been associated with damage to white matter, inflammation, and impaired blood-brain barrier permeability [26]. It has been reported that WMH is higher in patients with first-episode psychosis and schizophrenia [27]. A diffusion tensor imaging study found damage to the white matter, particularly in the anterior cingulate gyrus, in patients with OCD [28]. In another study, changes in white matter were reported in drug-naive OCD patients [29]. Subcortical WMH was reported in the case report of a treatment-resistant OCD patient [30]. According to the results of the present study, Fazekas grade 1 is more frequent in patients with OCD than healthy controls. This finding seems to be consistent with the findings of previous studies reporting white matter abnormalities in OCD patients [31]. The study’s results suggest an interesting association between Fazekas grades and YBOCS scores in OCD patients, indicating that the presence and severity of WMH in the brain may be linked to the severity of OCD symptoms. However, as with any research, it’s important for the findings to be independently verified and for further investigations to delve into the mechanisms and clinical implications of this association.

In this study, generalized cerebral atrophy was found to be significantly more common in OCD patients than in HC. There are studies in the literature reporting cortical thinning in OCD patients. Shin et al. reported that patients with OCD had thinner left inferior frontal, left middle frontal, left precentral, left superior temporal, left parahippocampal, left orbitofrontal, and left lingual cortices [32]. Nakamae et al. reported that the drug-naive OCD patients had statistically significant reduction in cortical thickness in the cluster that contained the left superior temporal gyrus and posterior insular cortex [33]. Additionally, OCD patients with cerebral atrophy had higher YBOCS scores and mean age. Cerebral atrophy is known to be more common in older individuals, and this observation aligns with that expectation. Cerebral atrophy refers to the loss of brain cells or a reduction in the size of the brain, which can be observed in brain imaging studies. It is a common feature associated with aging and can be a result of various neurological conditions.

This study’s lack of disease duration information limits the interpretation of the changes in the brain caused by OCD. Not looking at the family history of psychiatric illness prevents a more comprehensive look at the neurodevelopmental aspect. The lack of information about whether the patients are drug-naive or chronic is another shortcoming. Lastly, a study limitation is the absence of volumetric analysis other than morphological features.

## Conclusion

Based on this study, it is understood that CVI, a neurodevelopmental malformation, is more common in OCD patients. Due to the potential relationship of this anomaly with neuronal migration, it would be appropriate to pay attention to OCD symptoms in individuals with CVI and to perform white matter examination on brain imaging. In future studies, Fazekas grade can be evaluated in drug-naive OCD patients, and data on the pre-disease period can be obtained. Further studies may help to interpret these findings in OCD patients more comprehensively. In addition, the relationship between these findings and symptom severity in OCD can be investigated in the future.

## Data Availability

The datasets used and analyzed during the current study are available from the corresponding author on reasonable request.
